# High levels of macrolide-resistant *Mycoplasma genitalium* in Queensland, Australia

**DOI:** 10.1099/jmm.0.000584

**Published:** 2017-09-12

**Authors:** Ella Trembizki, Cameron Buckley, Cheryl Bletchly, Graeme R. Nimmo, David M. Whiley

**Affiliations:** ^1^​ UQ Centre for Clinical Research, The University of Queensland, Brisbane, Queensland 4029, Australia; ^2^​ Pathology Queensland Central Laboratory, Brisbane, Queensland 4029, Australia

**Keywords:** *Mycoplasma genitalium*, antimicrobial resistance, macrolide

## Abstract

The macrolide azithromycin is recommended for treatment of *Mycoplasma genitalium* infection; however, *M. genitalium* strains possessing macrolide resistance-mediating mutations (MRMMs) are increasingly being reported. Here, we used the SpeeDx ResistancePlus MG kit, which provides simultaneous detection of *M. genitalium* and MRMMs, to assess MRMM carriage among *M. genitalium* infections in Queensland, Australia. Performance characteristics of the ResistancePlus MG kit for *M. genitalium* detection were compared to in-house PCR. Available *M. genitalium* PCR-positive (*n*=67) and negative (*n*=281) samples from the years 2011 to 2017 were tested using the SpeeDx ResistancePlus MG kit. In total, 63.6 % *M. genitalium*-positive samples were indicated to harbour MRMMs. The ResistancePlus MG method provided sensitivity and specificity of 97 and 99.6 % respectively compared to in-house PCR for *M. genitalium* detection. Such high levels of macrolide-resistant *M. genitalium* raise further concerns over future use of azithromycin for treatment of *M. genitalium* infection.

*Mycoplasma genitalium* is a sexually transmitted infection that causes signs and symptoms which are similar to Chlamydia, including urethritis in males, and cervicitis and pelvic inflammatory disease in females [1]. Like Chlamydia, the macrolide azithromycin is the recommended first-line treatment for *M. genitalium* infection in most settings [2, 3]. However, treatment for *M. genitalium* is becoming problematic due to the high proportions of *M. genitalium* strains exhibiting resistance to the azithromycin caused by macrolide resistance-mediating mutations (MRMMs) in the *M. genitalium* 23S rRNA gene. The prevalence of *M. genitalium* strains harbouring MRMMs varies between geographical regions. For example, recent data showed MRMM-harbouring strains comprised 18 % of *M. genitalium* cases in Sweden [4] 47.3 % in Canada [5] and 72 % of cases in New Zealand [6]. Furthermore, prevalence may also differ between heterosexual populations and men-who-have-sex-with-men (MSM) with a recent study from Victoria, Australia (a southern state of Australia) showing that MSM were twice as likely to harbour MRMMs compared to heterosexual men (76 % compared to 39 %; [7]).

Antimicrobial resistance testing via bacterial culture is not feasible for *M. genitalium* as a routine tool given the fastidious nature of the organism. Therefore, nucleic acid amplification tests are now recommended for both diagnosing *M. genitalium* infection and assessing antimicrobial resistance [2]. The ResistancePlus MG assay (SpeeDx, Australia) is a PCR-based commercial method that simultaneously detects *M. genitalium* as well as five MRMMs [8]. Here, we used the ResistancePlus MG assay to investigate *M. genitalium* macrolide resistance levels in Queensland, Australia (a state in the northern part of Australia). In doing so, we also assessed the concordance of the ResistancePlus MG assay with the in-house PCR routinely used by the local pathology provider.

Testing was conducted using remnant DNA extracts from samples submitted to Pathology Queensland for routine *M. genitalium* investigation. Three convenience sample banks were included. Bank 1 comprised 33 *M. genitalium* PCR-positive samples from the period 2011–2013, whereas Bank 2 comprised 34 *M. genitalium* PCR-positive samples from the period 2016–2017 (Table 1). These 67 *M. genitalium* positive samples (combined numbers for Banks 1 and 2) were from the metropolitan regions of South East Queensland (primarily patients attending sexual health clinics in the Gold Coast and Brisbane), with one sample being from a regional area of Queensland. Bank 3 comprised 281 *M. genitalium* PCR-negative samples from both males (*n*=167) and females (*n*=114) in 2016, and included 65 cervical swabs, 124 urine samples, 28 vaginal swabs, 2 meatus swabs, 43 rectal swabs, 3 throat swabs, 6 urethral swabs, and 10 samples for which the site was not specified. Bank 1 samples were extracted using the using the High Pure Viral Nucleic Acid kit (Roche Diagnostics, Australia). Banks 2 and 3 comprised residual DNA extracts from the Cobas 4800 CT/NG test (Roche Diagnostics, Australia; for samples also being tested for *Neisseria gonorrhoeae* and *Chlamydia trachomatis*) or were otherwise extracted using the MagNA Pure instrument (Roche Diagnostics, Australia). The in-house PCR used by Pathology Queensland to screen the samples for *M. genitalium* used previously described primers and probe targeting the *M. genitalium* MgPa adhesin gene [9] and the QuantiTect Probe PCR Master Mix (Qiagen, Australia) as the basis for the reaction mix. All DNA extracts had been stored at −20 °C until being tested via the ResistancePlus MG method. ResistancePlus MG testing was performed as per kit instructions and results interpreted using the FastFinder ResistancePlus MG software (SpeeDx, Australia) following manufacturer’s instructions. Ethical approvals for the study were provided by the Human Research Ethics Committees of the Children’s Health Queensland Hospital and Health Service and the University of Queensland.

**Table 1.** Summary results for Banks 1 to 3

Sample bank	*M. genitalium* In-house PCR result	ResistancePlus MG		Specimen details	
		M. genitalium	23S rRNA mutation	Gender	Specimen site
Bank 1 (2011–2013)	33 POSITIVE	32 POSITIVE	21 POSITIVE	M=20	Urine (*n*=19) na (*n*=1)
				F=1	Urine (*n*=1)
			11 negative	M=10	Urine (*n*=9)
					Penile swab (*n*=1)
				F=1	Cervical swab (*n*=1)
		1 negative	Not assessable	F=1	Urine (*n*=1)
Bank 2 (2016–2017)	34 POSITIVE	33 POSITIVE	21 POSITIVE	M=19	Urine (*n*=16)
					Rectal swab (*n*=3)
				F=2	Vaginal swab (*n*=2)
			12 negative	M=6	Urine (*n*=5)
					Rectal swab (*n*=1)
				F=6	Vaginal swab (*n*=3)
					Cervical swab (*n*=1)
					Urine (*n*=1)
		1 negative	Not assessable	F=1	Vaginal swab (n=1)
Bank 3 (2016)	281 negative	1 POSITIVE	1 negative	M=1	Urine (*n*=1)
		280 negative	280 negative	M=166	Urine (n=108)
					Rectal swab (n=41)
					Urethral swab (n=6)
					Throat swab (n=3)
					Meatus swab (n=2)
					n/a (n=6)
				F=114	Cervical swab (n=65)
					Vaginal swab (n=28)

na, not available.

Table 1 provides a summary of all results. Briefly, when tested by the ResistancePlus MG assay, 32/33 (97.0 %) and 33/34 (97.1 %) of samples from Banks 1 and 2, respectively, provided positive results for *M. genitalium*. For Bank 3, one urine sample (0.4 %) was positive by ResistancePlus MG method (Table 1) and 280/281 (99.6 %) of *M. genitalium* in-house PCR-negative samples provided negative results. Based on the combined results of Banks 1 to 3, the ResistancePlus MG method provided a sensitivity of 97 % (65/67) and specificity of 99.6 % (280/281) compared to the in-house PCR for *M. genitalium* detection. For the total 66 samples from Banks 1, 2 and 3 providing positive results for *M. genitalium* by the ResistancePlus MG assay, 43 (63.6 %) were indicated to harbour 23S rRNA gene macrolide resistance mutations, including 21/32 (65.6 %) from 2011 to 2013 (Bank 1) and 21/34 (61.8 %) from 2016 to 2017 (Banks 2 and 3). The resistance levels from the two time periods were not significantly different (*P*=0.473) using Fisher’s exact test.

Overall, these data show high levels of macrolide-resistant *M. genitalium* in Queensland, Australia, and raise further concerns over azithromycin treatment of *M. genitalium* infections. The data also reinforce the importance of using a diagnostic assay to detect both the organism as well as resistance profile so as to facilitate selection of appropriate antimicrobials for treatment of *M. genitalium* infection.
